# Prehospital Emergency Ultrasound: A Review of Current Clinical Applications, Challenges, and Future Implications

**DOI:** 10.1155/2013/531674

**Published:** 2013-09-19

**Authors:** Mazen J. El Sayed, Elie Zaghrini

**Affiliations:** Department of Emergency Medicine, American University of Beirut Medical Center, P.O. Box 11-0236, Riad El Solh, Beirut 1107 2020, Lebanon

## Abstract

Imaging modalities in the prehospital setting are helpful in the evaluation and management of time-sensitive emergency conditions. Ultrasound is the main modality that has been applied by emergency medical services (EMS) providers in the field. This paper examines the clinical applications of ultrasound in the prehospital setting. Specific focus is on applications that provide essential information to guide triage and management of critical patients. Challenges of this modality are also described in terms of cost impact on EMS agencies, provider training, and skill maintenance in addition to challenges related to the technical aspect of ultrasound.

## 1. Introduction 

Emergency ultrasound performed by nonradiologists has been widely adopted in most emergency departments (EDs) across the United States (US) and the world with a continuously growing list of diagnostic and therapeutic applications [1]. This technology enables emergency physicians to answer focused clinical questions at the bedside, which would translate into faster and more accurate diagnosis and care of patients presenting with time-sensitive emergency conditions. Better outcomes have been reported with the use of emergency ultrasound [2].

The use of this technology in the prehospital setting is increasing with reports of physicians and nonphysicians performing diagnostic and therapeutic interventions in different emergency medical services (EMS) systems across Europe and the United States [3]. This was facilitated by the portability of modern ultrasound machines that have small, lightweight, and durable designs and that deliver high-quality and high-resolution imaging. 

Like any other intervention, the addition of ultrasound machines to the armamentarium of prehospital providers raises several questions in terms of potential clinical applications, feasibility, training requirements, cost, and more importantly its impact on the care process and on patient outcome. The type of EMS system that is in place, whether it follows the Anglo-American model or the Franco-German one, is an important factor to consider when discussing any prehospital intervention including prehospital ultrasound [4].

This paper reviews the available literature about current applications of ultrasound use in the prehospital setting and discusses challenges, limitations, and potentials of prehospital emergency ultrasound. The evidence presented is specific to emergency ultrasound performed in the prehospital setting and does not reflect the available evidence for all the medical indications and emergency or critical medicine recommendations for ultrasound use in the ED or in-hospital. 

## 2. Clinical Applications of Prehospital Ultrasound

Clinically relevant applications of emergency ultrasound in the prehospital setting fall into two broad categories: diagnostic and therapeutic. Most of the published literature of ultrasound use in the prehospital setting falls into the diagnostic category. 

Regardless of the type of EMS systems in terms of level of available prehospital providers, diagnostic applications, which can be easily learnt, can offer crucial information needed to guide the management of severely ill trauma and medical patients in the field and to help triage these patients to appropriate hospital destinations [5]. Therapeutic applications, on the other hand, are highly dependent on the sonographer's skill level or type of prehospital provider. 

### 2.1. Trauma Care

**Figure 1:**
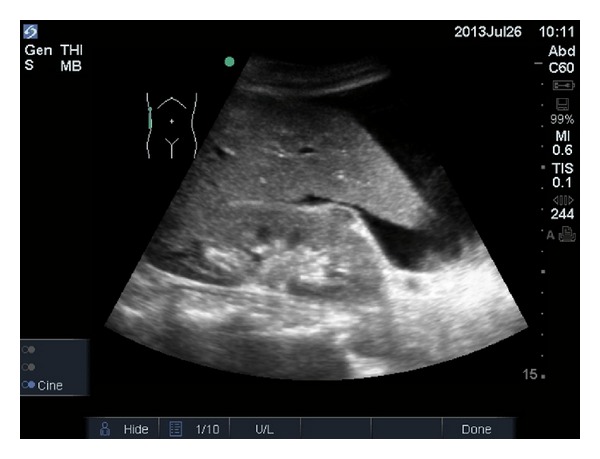
Free intra-abdominal fluid on ultrasound.

In EMS systems with regionalized trauma care and field triage guidelines [6] earlier detection of pericardial effusions in patients with penetrating thoracic trauma or of intra-abdominal free fluid (Figure 1) in patients with blunt trauma can be very helpful in helping providers decide on the method of transport and trauma center level destination.

In one prospective multicenter study of 202 trauma patients, prehospital focused abdominal sonography for trauma (PFAST) performed by emergency physicians and paramedics at the trauma scene had much higher sensitivity, specificity, and accuracy of detecting hemoperitoneum when compared to regular physical examination (93%, 99%, and 99%, resp., compared with 93%, 52%, and 57%). The PFAST examination time had a mean of 2–4 min (SD 0–8) and was completed 35 (SD 13) min prior to a regular emergency department (ED) FAST. A change in prehospital management, mainly fluid resuscitation, was reported in up to 21% of patients when PFAST was used. PFAST findings also influenced the decision making process regarding the mode of transport (ground versus helicopter) and the choice of hospital destination in up to one-third of patients [7]. In another study by Heegaard et al. trained paramedics carefully supervised by ultrasound-trained physicians detected free intraperitoneal or pericardial fluid in 7.1% of patients on whom FAST was performed in the prehospital field with excellent accuracy (100% proportion of agreement with physician overreader) [8]. Another published report also explored the potential of prehospital ultrasound to help rule out hemoperitoneum or hemopericardium in a trauma patient with PEA arrest [9]. 

Prehospital ultrasound use in trauma patients with suspected pneumothorax may be useful in preventing harm from unnecessary field intervention such as needle thoracostomy. When thoracic ultrasound was used to detect lung sliding sign (pleural sliding concomitant with insufflations or respirations in the absence of a pneumothorax) in the emergency department in patients after prehospital needle thoracostomy, 15 out of 57 (26%) trauma patients “appeared not to have had a pneumothorax originally nor to have had one induced by the needle thoracostomy” [10]. Harm to patients could potentially be avoided by the use of ultrasound prior to performing invasive procedures en route to hospital. 

Despite all the previous reports documenting improvement in diagnostic accuracy, a recent systematic review evaluating whether prehospital ultrasound improves treatment of trauma patients found that there is a lack of evidence regarding improved treatment [11]. 

### 2.2. Medical Care

Cardiac arrest and shock or prearrest conditions are other EMS priority conditions where prehospital ultrasound adds value to patient management and outcomes. 

In one prospective observational study of 230 patients in peri-resuscitation state (profound hypotension and/or severe dyspnea/tachypnea) or actively undergoing cardiopulmonary resuscitation (CPR), focused echocardiographic evaluation in life support (FEEL) performed in the prehospital setting altered the diagnosis and management in a significant number of patients [12]. The FEEL protocol was implemented by emergency physicians during an advanced-life-support (ALS-) conformed interruption of CPR of fewer than 10 s noting the following features: cardiac motion (present or absent), ventricular function (normal, moderately impaired, severely impaired, or absent), right ventricular dilatation, or pericardial collection [12]. In patients undergoing CPR, ultrasound use demonstrated cardiac wall motion in 13 out of 37 patients (35%) whose initial ECG diagnosis was asystole, which correlated with increased survival to hospital admission [12]. In addition to that, ultrasound helped, through detection of cardiac motion, differentiate between true PEA (TPEA or electromechanical dissociation) and pseudo-PEA (PPEA or coordinated electrical activity with no palpable pulse). PPEA was also associated with increased survival to hospital admission when compared with TPEA [12]. In patients in a peri-resuscitation state, ultrasound improved the diagnostic accuracy for potential diagnoses of tamponade, profound hypovolemia, myocardial insufficiency (severe left and/or right ventricular dysfunction), or thromboembolism (pulmonary or cardiac). These findings warranted a change in management in 89% of patients in the CPR group and 66% of patients in the peri-resuscitation group [12]. EMS systems with prehospital protocols that use asystole or PEA as criteria for field termination of the resuscitation can therefore benefit from adding ultrasound to such protocols [13, 14]. More evidence is, however, needed to rely solely on ultrasound findings to halt resuscitative efforts in patients with cardiac arrest. An observational study by Aichinger et al. examined the utility of prehospital emergency echocardiography in predicting outcomes in the management of cardiac arrest patients. Forty patients were included in their study. “Cardiac movement was associated with survival, and cardiac standstill at any time during CPR resulted in a positive predictive value of 97.1% for death at the scene” [15]. Their results did not support the use of prehospital ultrasound findings as the sole predictor of outcomes in cardiac arrest patients. A more recent systematic review by Blyth et al. examining whether detection of cardiac contractility on bedside echocardiography predicts return of spontaneous circulation (ROSC) during cardiac arrest reached the same conclusion [16].

 Prehospital ultrasound diagnostic applications have also been reported in patients with acute undifferentiated dyspnea. Prehospital ultrasound improves the accuracy of diagnosing pulmonary edema as the cause of acute dyspnea. In a prospective cohort study of 218 patients presenting with acute dyspnea (heart failure or COPD/asthma related), ultrasound performed by prehospital physicians in less than one minute, was found to be the strongest predictor for the diagnosis of heart failure in the prehospital setting [17]. Ultrasound was superior to both point-of-care N-terminal probrain natriuretic peptide testing and to clinical examination using Boston modified criteria [17, 18]. Seeing B-lines (sonographic artifacts caused by the interaction of water-rich structures and air) on the initial lung ultrasound had 100% sensitivity, 95% specificity, 100% negative predictive value, and 96% positive predictive value for the diagnosis of heart failure in the prehospital setting [17, 19].

Zechner et al. reported a similar benefit of improved accuracy in diagnosing the cause of acute dyspnea in two patients when prehospital ultrasound was used. This translated into improved clinical outcome when the treatment provided was based on the prehospital ultrasound findings [20].

Prehospital ultrasound was also reported to be useful in patients with unexplained hemodynamic instability where it helps differentiate between cardiac and noncardiac etiologies of shock. Adding ultrasound to prehospital shock management can help rule out the presence of life-threatening conditions such as clinically significant pericardial effusion or abdominal aortic aneurysms [12, 21, 22]. Boursier et al. also discussed the potential of prehospital ultrasound to detect massive pulmonary emboli (PE) in patients with refractory shock and when high clinical suspicion for PE exists [12, 23].

### 2.3. Airway Management

Another new diagnostic application of prehospital ultrasound consists of confirming endotracheal tube (ETT) placement through detection of the lung sliding sign [24]. Advanced airway management using ETT placement is commonly performed in EMS systems that employ ALS providers (paramedics) or physicians. End tidal CO_2_ capnography is the gold standard method for ETT correct placement confirmation. This method has some limitations in specific situations such as cardiac arrest, low cardiac output, acute pulmonary embolism, and hypothermia [25, 26]. Ultrasound offers prehospital providers an alternative method for ETT confirmation for recognizing tube displacement or differentiating between main tracheal intubation and right mainstem intubation. This technique is, however, operator dependent and is limited in the setting of pneumothorax or subcutaneous emphysema [24]. 

Most of the previously described diagnostic applications would help prehospital providers establish a more accurate diagnosis and guide patient management or triage to appropriate hospital destinations. On the other hand, therapeutic applications of ultrasound in the prehospital setting are highly dependent on the skill level and the scope of practice of the operator or prehospital provider. Applications such as ultrasound-guided pericardiocentesis have been described but mainly in systems with physicians working in the prehospital setting [12].

## 3. Challenges of Prehospital Ultrasound

Despite the wide range of applications for ultrasound in the prehospital setting, the adoption of this modality has been slow for several factors including, but not limited to, portability, cost, training and technical expertise of operators, and time limitations. Several studies with new handheld and portable models of ultrasound machines have demonstrated that ultrasound use is possible in most prehospital settings including land ambulances and helicopter EMS [5, 12, 27–29].

Time limitation is another challenge that is often cited as a reason for not using ultrasound. Lack of enough time was the main reason for not using thoracic ultrasound in one study examining the feasibility of thoracic ultrasound by HEMS [30]. Other studies have shown, however, that most focused ultrasound applications can be completed in less than 3 minutes without delaying the management or increasing on scene time [17, 28]. Even for the most time-sensitive conditions such as cardiac arrest, ALS compliant protocols of ultrasound use minimizing compression interruption time have been described and can be implemented in the prehospital setting [12, 31].

Training and technical expertise of providers are another challenge for ultrasound adoption in the prehospital setting. This limitation is pertinent only to EMS systems that use providers other than physicians to staff their ambulances. Physicians working in the prehospital setting would have to undergo the same training as other nonradiologist physicians who have ultrasound privileges and who use ultrasound in the ED or other settings (intensive care units, operating rooms). In EMS systems that use nonphysician providers such as the US and UK EMS systems, ultrasound is considered an advanced skill that is usually limited to advanced level providers such as paramedics. Ultrasound applications and more specifically therapeutic interventions are also closely tied to the scope of practice and skill levels of these nonphysician providers. 

Several studies have demonstrated that paramedics can easily acquire ultrasound skill with training duration varying from one hour and 15 minutes to two days depending on the type of diagnostic ultrasound application learned [7, 30, 32]. In one study by Roline et al. assessing the feasibility of thoracic ultrasound by HEMS flight crew, providers received a training in thoracic ultrasound consisting of a video of 15-minute duration, followed by hands-on session for 60 minutes to detect lung sliding sign. Forty-one patients underwent thoracic ultrasound with 54% of the images being considered to be of good quality. There was substantial agreement between the flight crew's interpretation and the expert reviewer's interpretation of the images (Cohen kappa statistic of 0.67 (95% CI, 0.44–0.90)) [30]. 

In another study by Chin et al., twenty emergency medical technicians paramedics with no prior ultrasonography training underwent training to acquire and recognize ultrasound images for several life-threatening conditions using the Prehospital Assessment with UltraSound for Emergencies (PAUSE) protocol [32]. The training consisted of 1 h lecture on the basics of ultrasonography, the PAUSE protocol, image acquisition, and basic image interpretation for the heart and lungs followed by one hour of hands-on session. When tested in a classroom setting, “paramedics obtained adequate images that could be used in evaluation of pneumothoraces, pericardial effusion, and cardiac standstill and correctly evaluated ultrasound video of those conditions” [32]. Higher success was documented for acquiring images to check for pneumothorax than for pericardial effusion or cardiac standstill [32].

In a different study by Heegaard et al. paramedics underwent 6 hours of structured ultrasound training and were able to adequately obtain and interpret prehospital FAST and abdominal aortic (AA) ultrasound images with 100% interpretation agreement with physician overreader [8]. Other published reports also support the successful training of paramedics in ultrasound use in the prehospital setting [33]. 

Initial ultrasound skill acquisition by paramedics is therefore possible with relatively short training courses. Ultrasound skill maintenance like any other skill requires practice and good quality management programs with physician oversight. 

One way to overcome the potential challenge of training prehospital providers to acquire and interpret ultrasound images is through the use of telesonography. Transmitting ultrasound images by different modalities from scene to ED is an effective tool that can be implemented in EMS systems that lack advanced level providers or in rural EMS systems for expert review of images and interpretation. Novel techniques of telesonography using cellular or satellite networks allow for the successful transmission of real-time ultrasound images from the prehospital setting to the ED without affecting the quality of the images [34]. 

The cost impact on EMS agencies introducing ultrasound into the prehospital setting has not been formally assessed. The new hand held ultrasound machines cost around US$ 9000. This is a significant cost for most systems especially when considering the number of units to be deployed in order to cover a large proportion of patients with time-sensitive emergency conditions on whom ultrasound use may reduce morbidity and improve outcomes. 

## 4. Future Implications

Acute ischemic stroke is another EMS priority condition that is time sensitive and where ultrasound is showing promise. In a recent study by Schlachetzki et al. 102 patients with acute stroke symptoms underwent prehospital transcranial color-coded sonography (TCCS) assessments [35]. Prehospital diagnosis of middle cerebral artery (MCA) occlusion using ultrasound was highly sensitive (90% (95% CI 55.5–99.75%) and specific 98% (95% CI 92.89–99.97%)) when compared to standard stroke imaging (CTA or MRA). The average time for completion of the ultrasound study by a neurologist was 5.6 min (SD 2.2). Field diagnosis was made early in the prehospital care phase (mean time to arrival of patient of 12.3 min (SD 7.09)) [35]. This study required, however, experienced neurologists who are skilled in neurosonography to perform the procedure and reach the diagnosis in a timely fashion. Future research regarding early ultrasound diagnosis of ischemic stroke in the prehospital setting should impact stroke management and improve on time to thrombolysis which would translate into better neurologic outcomes of stroke affected patients [36].

Prehospital ultrasound might have limited applications in the prehospital field that constitutes only one phase of emergency care. The scope of applications of emergency ultrasound is, however, much broader for emergency cases in other settings (ED, in-hospital, or remote areas). Added focus on three areas would increase the potential for ultrasound use in the prehospital field. First, enhancing the technology of telesonography for real-time assistance with interpretation of ultrasound images is important for EMS systems that lack advanced level providers. Second, developing effective ultrasound training programs for different level providers similar to the RUSH exam used by emergency physicians [37] is needed for timely evaluation and management of the critically ill in the field. Last but not least, dedicated research focusing on the benefit of performing existing clinical applications early in the field (abdominal aortic aneurysms in patients with abdominal pain) would support the use of prehospital ultrasound and potentially improve patient outcomes. 

## 5. Conclusion

Prehospital emergency ultrasound has many clinical applications that would reduce morbidity and improve outcomes of patients with life-threatening emergency conditions. This imaging modality improves diagnostic accuracy and provides crucial information to prehospital providers to guide management and help triage patients to appropriate destinations. Training requirements and time limitations are the main challenges to prehospital ultrasound utilization. Structured training of nonphysician prehospital providers is needed to provide them with adequate ultrasound skill acquisition and maintenance. The potential for use of this modality in the prehospital setting is great; however outcome research is needed to provide stronger evidence on its clinical impact on patient outcome.

## References

[1] Goodman TR, Scoutt LM, Brink JA (2011). A survey of emergency physician-performed ultrasound: implications for academic radiology departments. *Journal of the American College of Radiology*.

[2] Plummer D, Brunette D, Asinger R, Ruiz E (1992). Emergency department echocardiography improves outcome in penetrating cardiac injury. *Annals of Emergency Medicine*.

[3] Nelson BP, Chason K (2008). Use of ultrasound by emergency medical services: a review. *International Journal of Emergency Medicine*.

[4] Garrone M (2011). Prehospital ultrasound as the evolution of the Franco-German model of prehospital EMS. *Critical Ultrasound Journal*.

[5] Lapostolle F, Petrovic T, Lenoir G (2006). Usefulness of hand-held ultrasound devices in out-of-hospital diagnosis performed by emergency physicians. *American Journal of Emergency Medicine*.

[6] Sasser SM, Hunt RC, Faul M (2012). Guidelines for field triage of injured patients recommendations of the national expert panel on field triage, 2011. *Morbidity and Mortality Weekly Report*.

[7] Walcher F, Weinlich M, Conrad G (2006). Prehospital ultrasound imaging improves management of abdominal trauma. *British Journal of Surgery*.

[8] Heegaard W, Hildebrandt D, Spear D, Chason K, Nelson B, Ho J (2010). Prehospital ultrasound by paramedics: results of field trial. *Academic Emergency Medicine*.

[9] Polk JD, Fallon WF (2000). The use of focused assessment with sonography for trauma (FAST) by a prehospital air medical team in the trauma arrest patient. *Prehospital Emergency Care*.

[10] Blaivas M (2010). Inadequate needle thoracostomy rate in the prehospital setting for presumed pneumothorax: an ultrasound study. *Journal of Ultrasound in Medicine*.

[11] Jørgensen H, Jensen CH, Dirks J (2010). Does prehospital ultrasound improve treatment of the trauma patient? A systematic review. *European Journal of Emergency Medicine*.

[12] Breitkreutz R, Price S, Steiger HV (2010). Focused echocardiographic evaluation in life support and peri-resuscitation of emergency patients: a prospective trial. *Resuscitation*.

[13] Morrison LJ, Visentin LM, Kiss A (2006). Validation of a rule for termination of resuscitation in out-of-hospital cardiac arrest. *The New England Journal of Medicine*.

[14] Kellermann AL, Hackman BB, Somes G (1993). Predicting the outcome of unsuccessful prehospital advanced cardiac life support. *Journal of the American Medical Association*.

[15] Aichinger G, Zechner PM, Prause G (2012). Cardiac movement identified on prehospital echocardiography predicts outcome in cardiac arrest patients. *Prehospital Emergency Care*.

[16] Blyth L, Atkinson P, Gadd K, Lang E (2012). Bedside focused echocardiography as predictor of survival in cardiac arrest patients: a systematic review. *Academic Emergency Medicine*.

[17] Prosen G, Klemen P, Štrnad M, Grmec S (2011). Combination of lung ultrasound (a comet-tail sign) and N-terminal pro-brain natriuretic peptide in differentiating acute heart failure from chronic obstructive pulmonary disease and asthma as cause of acute dyspnea in prehospital emergency setting. *Critical Care*.

[18] Remes J, Miettinen H, Reunanen A, Pyorala K (1991). Validity of clinical diagnosis of heart failure in primary health care. *European Heart Journal*.

[19] Rempell JS, Noble VE (2011). Using lung ultrasound to differentiate patients in acute dyspnea in the prehospital emergency setting. *Critical Care*.

[20] Zechner PM, Aichinger G, Rigaud M, Wildner G, Prause G (2010). Prehospital lung ultrasound in the distinction between pulmonary edema and exacerbation of chronic obstructive pulmonary disease. *American Journal of Emergency Medicine*.

[21] Ward DI (2007). Prehospital point-of-care ultrasound use by the military. *Emergency Medicine Australasia*.

[22] Brun PM, Chenaitia H, Gonzva J, Bessereau J, Bobbia X, Peyrol M (2013). The value of prehospital echocardiography in shock management. *The American Journal of Emergency Medicine*.

[23] Boursier F, Maistre JP, Saddedine M, Pernot T, Adnet F (2004). Prehospital thrombolysis of a pulmonary embolism with a severe shock. *Annales Francaises d’Anesthesie et de Reanimation*.

[24] Brun PM, Besserau J, Cazes N, Querrellou E, Chenaitia H (2012). Lung ultrasound associated to capnography to verify correct endotracheal tube positioning in prehospital. *The American Journal of Emergency Medicine*.

[25] Grmec Š (2002). Comparison of three different methods to confirm tracheal tube placement in emergency intubation. *Intensive Care Medicine*.

[26] Takeda T, Tanigawa K, Tanaka H, Hayashi Y, Goto E, Tanaka K (2003). The assessment of three methods to verify tracheal tube placement in the emergency setting. *Resuscitation*.

[27] Price DD, Wilson SR, Murphy TG (2000). Trauma ultrasound feasibility during helicopter transport. *Air Medical Journal*.

[28] Busch M (2006). Portable ultrasound in pre-hospital emergencies: a feasibility study. *Acta anaesthesiologica Scandinavica*.

[29] Snaith B, Hardy M, Walker A (2011). Emergency ultrasound in the prehospital setting: the impact of environment on examination outcomes. *Emergency Medicine Journal*.

[30] Roline C, Heegaard W, Moore J (2013). Feasibility of bedside thoracic ultrasound in the helicopter emergency medical services setting. *Air Medical Journal*.

[31] Niendorff DF, Rassias AJ, Palac R, Beach ML, Costa S, Greenberg M (2005). Rapid cardiac ultrasound of inpatients suffering PEA arrest performed by nonexpert sonographers. *Resuscitation*.

[32] Chin EJ, Chan CH, Mortazavi R (2013). A pilot study examining the viability of a Prehospital Assessment with UltraSound for Emergencies (PAUSE) protocol. *The Journal of Emergency Medicine*.

[33] Brooke M, Walton J, Scutt D (2010). Paramedic application of ultrasound in the management of patients in the prehospital setting: a review of the literature. *Emergency Medicine Journal*.

[34] Ogedegbe C, Morchel H, Hazelwood V, Chaplin WF, Feldman J (2012). Development and evaluation of a novel, real time mobile telesonography system in management of patients with abdominal trauma: study protocol. *BMC Emergency Medicine*.

[35] Schlachetzki F, Herzberg M, Hölscher T (2012). Transcranial ultrasound from diagnosis to early stroke treatment—part 2: prehospital neurosonography in patients with acute stroke—The regensburg stroke mobile project. *Cerebrovascular Diseases*.

[36] Hölscher T, Dunford JV, Schlachetzki F (2013). Prehospital stroke diagnosis and treatment in ambulances and helicopters-a concept paper. *The American Journal of Emergency Medicine*.

[37] Perera P, Mailhot T, Riley D, Mandavia D (2010). The RUSH exam: rapid Ultrasound in SHock in the evaluation of the critically lll. *Emergency Medicine Clinics of North America*.

